# Pulmonary Biomarkers of Bronchopulmonary Dysplasia

**DOI:** 10.4137/bmi.s834

**Published:** 2008-07-02

**Authors:** Alecia Thompson, Vineet Bhandari

**Affiliations:** Department of Pediatrics, Division of Perinatal Medicine, Yale University School of Medicine, New Haven, CT 06520

**Keywords:** cytokines, lung, premature neonate

## Abstract

Bronchopulmonary dysplasia, or BPD, is a chronic pulmonary disorder of premature infants, commonly defined as having an oxygen requirement at 36 weeks postmenstrual age. It is an important source of morbidity and mortality in premature neonates. Its’ etiology appears to be multifactorial with the most common associations being prematurity, need for mechanical ventilation, and oxygen exposure. Implied in the pathogenesis of BPD is the role of cytokines which are immune mediators produced by most cell types. This is evidenced by studies in which there exist alterations in the levels of “pro-inflammatory” and “anti-inflammatory” cytokines. The imbalance of these cytokines have either heralded the onset or predicted the presence of BPD, or indicated a decreased propensity to developing this chronic respiratory disorder of preterm infants. Many other pulmonary markers have been shown to be altered in patients with BPD. These include markers indicative of altered lung repair processes, decreased endothelial integrity, oxidative damage and abnormal fibrinolytic activity, all of which are thought to be mechanisms contributing to the development of BPD.

In this review, we will discuss the physiologic role of specific biomarkers in the pulmonary tract of the human premature neonate, the perturbations that enable them to be deranged, and their proposed association with BPD.

## Introduction

Bronchopulmonary dysplasia, or BPD, is a chronic pulmonary disorder of premature infants and is commonly defined as having an oxygen requirement at 36 weeks postmenstrual age. It is an important source of morbidity and mortality in premature neonates. Overall, 30% of premature neonates with a birth weight less than 1500 grams are affected, but the majority of these are neonates with a birth weight of 501–750 grams, who have BPD at a rate of 52%. The multifactorial etiology of BPD includes genetic predisposition, baro-trauma and volu-trauma from mechanical ventilation in surfactant-deficient premature lungs, reactive oxygen species from prolonged oxygen use and high oxygen concentrations, pre- and postnatal infections, and presence of a patent ductus arteriosus (PDA) with its’ resultant pulmonary over-circulation.(1, 2) The aforementioned factors and the relatively poor, early postnatal nutrition of premature infants promote abnormal postnatal development of the immature lung. These factors contribute to inflammation, damage, attenuated repair and growth of gas-exchanging surfaces, leading to the clinical manifestations inherent to BPD.

Cytokines are immune mediators produced by a variety of cell types, and have been implicated in the pathogenesis of BPD. This is evidenced by studies in which there exist alterations in the levels of “pro-inflammatory” and “anti-inflammatory” cytokines. The imbalance of these cytokines have heralded the onset, predicted the presence of BPD, or indicated a decreased propensity to developing this chronic respiratory disorder of preterm infants. Other biomarkers measured in tracheal fluid have been altered in patients at risk for, or diagnosed with BPD. These include markers indicative of altered lung repair processes, decreased endothelial integrity, oxidative damage and decreased fibrinolytic activity, all factors potentially contributing to the pathogenesis of BPD.

## Lung Development

Intrinsic to the development of BPD is prematurity. Normal lung development occurs in phases which correspond to structural differences. The first phase, the embryonic phase, occurs at 3–7 weeks post-menstrually. The pseudoglandular phase occurs next, from 5–17 weeks. At 16–26 weeks postmenstrual age, the cannalicular phase occurs. Many premature neonates may be born during this phase of lung development. The saccular phase occurs at 24–38 weeks and accounts for the time when the majority of patients who develop BPD are born. The last phase, the alveolar phase, occurs from about 32 weeks postmenstrual age, till about 18 months after birth, with the majority of the alveolarization process occurring 5–6 months after birth.(3) Intrinsic to the process of normal lung development is alveolarization, which incorporates the processes of “elastogenesis and angiogenesis,” with multiple cell to cell interactions between epithelial cells (type 1 and 2 pneumocytes), fibroblasts, interstitial cells and endothelial cells.(4) Premature birth, with its subsequent medical disorders and their management, alters the postnatal development of these very premature lungs, via changes in the usual signaling pathways. Factors that disrupt normal angiogenesis, control of inflammation (i.e. imbalance between “pro”- and “anti-inflammatory” cytokines), and appropriate fibrin depostion or removal are at play in the pathogenesis of BPD. Many of the pulmonary biomarkers that have been studied relate to the possible disruption in the above mechanisms.

## Pathophysiology of BPD

### Clinical manifestations and treatment

Clinically, BPD presents as continued oxygen dependence, observed as a premature infant approaches 36 weeks postmenstrual age. The pharmacologic armamentarium may include diuretics, postnatal steroids and caffeine. Occasionally beta-2 agonists, inhaled anticholinergics and vitamin A are used.(1) Other strategies include fluid restriction, adequate caloric intake to enhance growth and healing, and gentle ventilatory support as needed.

### Radiographic and pathologic findings

Recently there has been a change in the radiographic and pathologic findings associated with BPD. This change has likely been brought about by the younger gestational age of surviving infants, widespread use of surfactant, and less aggressive respiratory management strategies. The result has been a change in the architectural aberrations that are noted in the lungs of premature neonates with BPD. Traditionally, in the pre-surfactant era, BPD or chronic lung disease of prematurity, was represented on radiographic imaging by emphysematous areas with other portions of the lung displaying volume loss. Pathologically, pulmonary specimen would also exhibit signs of increased inflammation, fibrosis and small airway disease. Pathologic findings of more recent BPD include impaired alveolar and pulmonary vascular development, with dilated alveolar spaces, and minimal alveolar fibrosis and inflammation when compared to the older form of BPD. Radiographically, the lungs initially appear hazy, dense, and progress to hyperinflation, but without the large cysts typical of the older form of BPD.(2, 5, 6)

### Role of cytokines

Cytokines are chemicals produced by virtually all cell types including white blood cells, endothelial and epithelial cells, fibroblasts and type II pneumocytes. They mediate immune, inflammatory and hematopoietic functions in response to various stimuli.(7) In preterm infants, inciting events cause an inflammatory response, either systemically or localized to the pulmonary organ. We have only a partial understanding of how inflammation in the setting of prematurity causes BPD. It likely results from an imbalance of “pro”- and “anti-inflammatory” factors. Soon after birth, upon initiation of mechanical ventilation, there is an influx of neutrophils and macrophages into pulmonary interstitium. Neutrophils adhere to the endothelium, with interaction between these two cell types occurring via adhesion molecules (e.g. selectins and integrins). This allows for extravasation of neutrophils and macrophages towards the specific areas of injury. These inflammatory cells produce cytokines and other signaling molecules for augmentation of the inflammatory response in an attempt to mitigate the damage of the inciting insult. There is subsequent disruption of the alveolar capillary unit and pulmonary tissue integrity. The imperfect attempt at repair, differentiation and growth of these tissues act in concert with the inflammation to produce the biochemical profile, signs and symptoms we see in patients with, and those at risk, for BPD.(2, 3, 8, 9) Many studies have been conducted to evaluate the presence of cytokines in the amniotic fluid, cord blood, plasma and tracheal secretions of preterm neonates at risk or who have BPD in an effort to determine a biochemical profile of the condition.

### Proposed mechanisms

**Figure 1:**
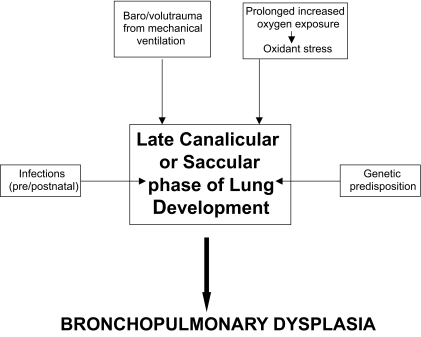
Multifactorial etiology of Bronchopulmonary Dysplasia.

**Figure 2:**
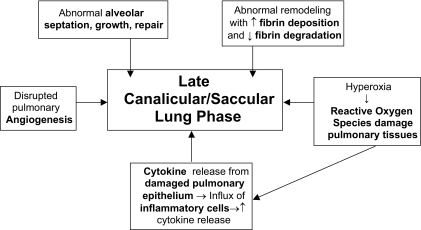
Sources of pulmonary biomarkers of Bronchopulmonary Dysplasia.

Genetic predisposition, baro-trauma and volu-trauma from mechanical ventilation in surfactant-deficient premature lungs, reactive oxygen species from prolonged oxygen use and high oxygen concentrations, pre- and postnatal infections, and presence of a PDA have all been factors associated with the development of BPD.(1, 5, 9, 10) See Figures 1 and 2.

#### Genetics

In a paper published in 2006, Bhandari and colleagues explored the genetic basis for conditions common to very premature infants. BPD and its’ potential for genetic predisposition was assessed. It was observed that in premature twins (63 monozygotic and 189 dizygotic twin pairs), less than 32 weeks gestational age, BPD occurred at a rate of 29% in one or more of a twin pair. After controlling for other covariates, 53% (p = 0.004) of BPD was explained by genetic factors alone.(11) Multiple genetic variants have been assessed in the evaluation of carrier states that predispose to the development of BPD. The interested reader is directed toward a recent review of this topic for more detailed information.(12)

#### Prenatal infections

Chorioamnionitis may be a risk factor for the development of BPD. Though the pathogenesis is incompletely understood, multiple studies have studied the cytokine presence in the amniotic fluid of infants born prematurely, for reasons which may include chorioamnionitis. Some studies have indicated increased levels of cytokines, predominantly interleukins (IL), IL-1β, IL-6, IL-8, and many of these patients go on to develop BPD.

Though there is difficulty in correlating clinical and histologic chorioamnionitis, it has been shown that those infants who are born prematurely before 28 weeks postmenstrual age have evidence of histologic chorioamnionitis, and have worse pulmonary outcomes at a higher rate than those born at later gestational ages. They also had more elevated levels of inflammatory cytokines in their blood.(13) Watterberg and colleagues have reported that infants exposed to intrauterine inflammation have decreased rates of respiratory distress syndrome (RDS); however, they had no decrease in rates of BPD.(14) Other authors have explored the relationship between chorioamnionitis and BPD and have indicated that inutero exposure to low level inflammation may induce a pulmonary maturation, possibly leading to decreased rates of BPD.(10) Van Marter and colleagues found that antenatal infection, coupled with postnatal sepsis, and mechanical ventilation increased the rates of BPD in preterm neonates.(15)

#### Postnatal infections

Systemic infections such as sepsis, necrotizing enterocolitis, etc., predispose to the development of BPD. The inflammatory response occurring secondary to infection, increases release of inflammatory and vasoactive cytokines on an already damaged pulmonary organ, increasing the need for mechanical ventilation. Airway colonization, with increased neutrophilic presence and “pro-inflammatory” cytokines may also predispose to the development of BPD.(2) Other studies have indicated a predisposition to BPD in neonates with postnatal infections.(15)

#### Baro- and Volutrauma

Mechanical ventilation has long been associated with the development of BPD. Intubated neonates who ultimately develop BPD often have significantly increased levels of “pro-inflammatory” cytokines, possibly from microbial colonization and increased distention of alveolar tissue with subsequent baro- and volutrauma leading to increased inflammation.(2, 3, 5, 16)

The association of presence of a PDA and BPD is observed likely due to the increase in pulmonary circulation and subsequent need for ventilatory support and oxygen administration, factors leading to BPD development.

#### Hyperoxia

Excessive oxygen induces oxidative stress with production of reactive oxygen species which directly damages respiratory epithelium by inducing necrosis and/or apoptosis, subsequently increasing production of inflammatory cytokines.(17, 18) Initial hyperoxic cellular damage induces alveolar or interstitial macrophages to express early cytokines such as IL-1 and tumor necrosis factor (TNF). Chemoattractants are then induced from multiple cell types in the lung, in turn recruiting other inflammatory cells, such as neutrophils.(18)

## Source of Biomarkers in BPD

Though the biomarkers indicating a likelihood of developing BPD may be measured in serum, our interest lay in studies that assessed their pulmonary concentrations specifically. It was felt that this would give a more precise representation of the pathological processes specific to the lung and decrease the possibility that altered levels of biomarkers existed only because there was systemic pathology, or that which involved another organ. Bronchoalveolar lavage is one such procedure that may yield accurate information, but is deemed more invasive than performing tracheal aspirates. In addition, D’Angio and colleagues have shown that tracheal aspirates when done under standardized technique in neonates can be a suitable alternative to bronchoalveolar lavage in most circumstances.(19) One could postulate that these methods of sampling may be more representative of responses more proximal in the respiratory tract and not accurately reflect the processes present in the human premature lung that indicate or predispose to BPD. However, the routine and relatively standardized performance of obtaining tracheal aspirates from intubated neonates makes it an accessible option for the evaluation of pulmonary biomarkers. Hence, the majority of the pulmonary biomarkers to be discussed in the next section have been measured in tracheal aspirates.

## Pulmonary Biomarkers in BPD

**Figure 3:**
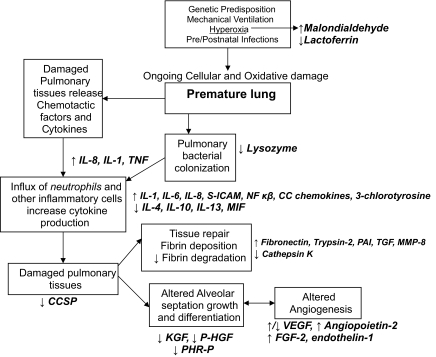
Proposed sequence of events eliciting alterations in the pulmonary biomarkers observed in Bronchopulmonary Dysplasia (see text for abbreviations).

Though there are many clinical associations indicative of an increased risk for the eventual development of BPD, currently there exists no one factor or marker that uniformly and accurately predicts its development. We are limited to waiting for oxygen dependence at 36 weeks postmenstrual age to determine its’ presence. The inciting events that predispose to development of BPD likely occur fairly soon after birth, and we are limited by a lack of ability to distinguish very early on, those neonates who will likely go on to develop this chronic disorder. All the biomarkers below are likely abnormal due to damage that has already occurred. See Figure 3.

Some biomarkers are capable of being perceived as either “pro-inflammatory” or anti-inflammatory,” depending on their concentration relative to their physiologic role.

Categories of pulmonary biomarkers that are deranged in BPD are interleukins and other chemo-attractant molecules, markers indicative of abnormal angiogenesis, those signifying abnormal fibrin deposition or degradation, markers of oxidative damage, and peptide growth factors. These have been summarized in Table 1.

### Interleukins (IL)

Interleukins are chemicals made by one cell type that act on other inflammatory cells. They may induce growth, differentiation and proliferation of other leukocytes. They may also act as chemo-attractants for recruitment of other cell types (immune and non-immune cells) to a site of injury, and may exert pro- or anti-inflammatory effects.

### IL-1β, 6, 8, 16

IL-1β and 6 are particularly active in the acute phase response to injury, while IL-8 is a potent chemotactic agent for recruitment of neutrophils. Typically viewed as proinflammatory, these cytokines have been shown to be elevated very early in the respiratory course of the preterm population that ultimately develops BPD, and in those with BPD at the time their tracheal aspirates are assessed. These findings have been replicated in multiple studies.(20–27)

IL-16 acts as a chemoattractant for CD4+ T lymphocytes, monocytes and eosinophils. In a study to correlate levels of pro-inflammatory cytokine IL-16 and its’ relation to BPD, Wang and colleagues, enrolled 34 intubated infants, 27 of whom were preterm. BPD developed in 11 of the preterm infants, with 6 of the 11 infants having detectable IL-16 levels, i.e. 16 of 46 tracheal aspirate samples. This was compared to 1 of 30 tracheal samples from non-BPD patients positive for IL-16, p = 0.001. Levels of IL-16 also correlated significantly with neutrophil counts in the tracheal aspirates.(28)

### IL-10, 4, 13

These cytokines usually act in an anti-inflammatory capacity, and are responsible for the production, differentiation and proliferation of B cells and macrophages. One could hypothesize that decreased levels of “anti-inflammatory” cytokines would predispose to the development of BPD given the role that inflammation likely has in this disorder. However, there are discrepant findings regarding the levels of “anti-inflammatory” cytokines in the tracheal fluid of preterm infants at risk for BPD, particularly IL-10, seemingly the most studied.

#### IL-10

In Vento and colleagues’ study to assess the levels of seven cytokines and their association with the development of BPD, it was found that IL-10 was decreased in those infants who developed the disorder, on days 1, 3 and 5.(22) Oie, et al.’s study in 48 preterm infants, with tracheal aspirate samples taken in the first seven days of life, also confirmed this finding.(29) McColm and colleagues found detectable levels of IL-10 in tracheal aspirates of preterm ventilated infants, but could not correlate the levels with development of BPD.(30) Garingo and colleagues, attempted to induce production of IL-10 from lung inflammatory cells (obtained from infants, 33/37 who were premature), with lipopolysaccharide (LPS), a potent inducer of inflammation. Their results indicated a decreased ability to induce IL-10 in those infants who went on to develop BPD; i.e. 97% of the preterm infants unable to constitutively express the IL-10 gene developed BPD.(31)

#### IL-4, 13

IL-4 is produced by T lymphocytes and alveolar macrophages, and induces cytokines, some “pro-inflammatory” and others “anti-inflammatory”. However, they are typically felt to be mostly anti-inflammatory in their actions. Overall, in a study by Baier and colleagues to assess levels of IL-4 and IL-13, it was found that levels of these potentially “anti-inflammatory” cytokines could not be significantly correlated with the development of BPD.(32)

### Angiopoietin-2

Angiopoietin-2 is an angiogenic growth factor that destabilizes blood vessels, enhances vascular leak and induces endothelial cell necrosis in hyperoxic conditions. Its’ concentration was observed to be increased in infants with BPD and/or death, when compared to those infants with RDS who recovered.(17, 33)

### Cathepsin K

Cathepsin K is a cysteine protease, which degrades existing extracelluar matrix and regulates the release of matrix proteins from fibroblasts. In one study, 13 intubated patients with 46 tracheal aspirates were evaluated. Six of 13 infants developed BPD, and had declining cathepsin K levels by day13, when compared to neonates who did not.(34)

### CC Chemokines

There are 4 families of chemokines (chemotactic cytokines); they play a role in regulating inflammation by recruiting inflammatory cells to the area of injury. The ‘CC’ family consists of monocyte chemoattractant proteins (MCP) 1, 1α, 1β, 2 and 3. These chemokines were measured in a study to assess the role of CC chemokines in acute lung injury in the preterm, ventilated infant over the first 21 days of life. Fifty-six very low birth weight infants were enrolled, and the above chemokines were measured. Fourteen infants developed BPD. MCP- 1, 1α, 1β, 2 and 3 were all increased in those infants developing BPD. However, only MCP- 1, 2 and 3 were statistically significant, with MCP-3 being the most significant.(35)

### Clara cell secretory protein (CCSP)

CCSP is produced by Clara cells, which are non-ciliated epithelial cells lining the respiratory and terminal bronchioles. It is hypothesized that this protein may act to modulate acute pulmonary inflammatory processes. In 24 preterm infants with 98 tracheal aspirate samples taken up to 2 weeks after birth, it was noted that Clara cell secretory protein increased with maturity, and was increased in those neonates who had evidence of infection.(36) In another study by Ramsay and colleagues, they enrolled 45 preterm neonates, with 19 developing BPD. Their hypothesis was that oxidation of proteins important to pulmonary function occurred and predisposed preterm infants to BPD. Overall their results indicated that lower levels of CCSP predisposed to development of BPD.(37)

### 3-Chlorotyrosine

3-Chlorotyrosine is a biomarker of the neutrophil oxidant, hypochlorous acid, used in the inflammatory process. Its’ only source is from neutrophils and monocytes which release myeloperoxidase to catalyze oxidation of chloride by hydrogen peroxide to give hypochlorous acid. In support of data that neutrophilic infiltration and subsequent oxidative injury is closely related to the development of BPD, Buss and colleagues assessed levels of 3-chlorotyrosine, and found that an increase in their concentration correlated with development of BPD.(38)

### Fibronectin

An extracellular matrix component, fibronectin is important in maintaining the integrity of pulmonary tissues and microvasculature. Watts and colleagues enrolled 32 infants in a study to assess fibronectin levels in those infants developing BPD. Eighteen out of 32 developed BPD. Their results indicate a higher fibronectin level in those infants developing BPD.(39)

### Lactoferrin and Lysozyme

Lactoferrin is hypothesized to be a marker of inflammation by regulating granulocyte and macrophage proliferation, and may also have some antioxidant properties. Lysozyme is a bactericidal protein that degrades the walls of susceptible bacteria. Both are hypothesized to be released from the serous cells of the submucous glands of the respiratory tract. Revenis and colleagues studied 36 preterm patients, of which 18 developed BPD. It was hypothesized that the levels of neutrophil-derived lactoferrin and lysozyme would be increased in the airway fluid from babies “predisposed” to the development of BPD. Both lactoferrin and lysozyme when measured in the first 3 days of life were decreased in those patients with BPD as compared to those who did not have BPD, with the decrease in lysozyme being significant.(40)

### Macrophage migration inhibitory factor (MIF)

MIF is an upstream regulator of the innate immune response. It has been implicated in the pathogenesis of a number of inflammatory disorders including sepsis, acute respiratory distress syndrome (in adults), asthma, and inflammatory/autoimmune diseases. MIF was quantified in tracheal aspirates obtained during the first 2 days of life in a cohort of 26 neonates with RDS. There was a reduction in the concentration of MIF in the lungs of those infants with RDS who went on to develop BPD.(41)

### Malondialdehyde

Malondialdehyde is a marker of oxidative damage, possibly from oxygen radicals produced under hyperoxic conditions, or the respiratory burst from inflammatory cells present in the pulmonary organ. In one study of 43 preterm ventilated infants, its’ pulmonary concentrations were noted to be elevated. However, this elevation in the concentration of malondialdehyde was only weakly correlated with the development of BPD.(42)

### Matrix metalloproteinase-8 (MMP-8)

Matrix metalloproteinases are a family of endoproteinases that aid in the remodeling and degradation of extracellular matrix and basement membranes. They are secreted in inactive form, activated in extracellular spaces and cell surfaces by oxidants and serine proteinases. They also have the capacity to activate each other. The development of BPD is partially characterized by disordered pulmonary repair after inflammation. Cederqvist, et al. found that patients who went on to develop BPD had increased levels of tracheal MMP-8, with decreased levels of its’ inhibitor ‘tissue inhibitor of metalloproteinases’ (TIMP).(43)

### Nuclear factor- kappa B (NF-κβ)

NF-κβ is a transcription factor activated by signs of cellular stress, subsequently promoting expression of multiple genes including pro-inflammatory cytokines. Tracheal aspirates of thirty-three preterm infants were assessed several times over the first 2 weeks of life, for correlation between NF***-***κβ levels and development of BPD. Levels of this biomarker were significantly elevated in the infants who developed BPD or died.(44)

### Parathyroid hormone-related protein (PTHrP)

PTHrP is secreted by type II pneumocytes and plays a role in the normal alveolar growth and development. It’s signaling was shown to be down-regulated in alveolar over-distention and under hypoxic conditions.(45, 46) It was then thought that PTHrP levels would correlate with development of BPD. In 2006, Rehan and colleagues, studied this by enrolling 40 VLBW infants; 12 of 40 developed BPD, and had significantly lower levels of PTHrP in the first week of life, when compared to those who did not.(47)

### Plasminogen activator inhibitor-1 (PAI-1)

PAI-1 is a regulator of fibrinolysis. It was hypothesized that inhibition of fibrinolysis may play a role in the development of BPD. Thirty-seven intubated, preterm infants were enrolled and tracheal aspirates taken in the first two weeks of life. Fifteen infants developed BPD with significantly higher levels of PAI-1 than non-BPD patients.(48)

### Pulmonary trypsin-2

Trypsin-2 is a serine protease expressed in a variety of human epithelial cell types, including the lung. It can directly attack extracellular protein matrix, basement membrane proteins, and can activate matrix metalloproteinases (MMPs) and initiate protease cascades, causing tissue destruction. It was hypothesized by Cederqvist and colleagues that when trypsin is ineffectively inhibited by its’ natural inhibitor tumor-associated trypsin inhibitor (TATI), it may play a role in the development of BPD. Thirty-nine infants were studied in the first 2 weeks of life, yielding 249 tracheal aspirate samples for evaluation. Results indicated that there was significantly higher trypsin-2 to TATI ratio during weeks one and two in those infants developing BPD.(49)

### Soluble intercellular adhesion molecule-1 (ICAM-1)

Intercellular adhesion molecule-1 plays a role in early inflammation and is the ligand for lymphocyte function-associated antigen 1 (LFA-1). ICAM-1 is expressed by mast cells, lymphocytes, eosinophils, endothelial and bronchial epithelial cells. Tracheal aspirates from 15 premature neonates were analyzed for soluble ICAM-1 content and evaluated for the relation to the development of BPD. Nine infants developed BPD, and it was found that these patients had significantly higher levels of soluble ICAM-1 at 6–14 days of age.(50)

### Transforming growth factor–β1 (TGF–β1)

TGF–β1 is produced mostly by alveolar macrophages, and acts on fibroblasts to increase the transcription of fibronectin and procollagen. It also inhibits synthesis of proteases and increases the synthesis of anti-proteases, thus resulting in a net increase in fibrosis. In a study to assess the role of TGF-β1 in the development of BPD, 40 preterm infants were enrolled. In this study, BPD was defined as oxygen dependency at 28 days with ‘radiographic changes.’ Eighteen infants developed BPD and had significantly increased levels of TGF-β1 in tracheal aspirates, when compared to those with RDS and the controls.(51) TGF-β1 was also noted to be increased in one study assessing the levels of seven cytokines in ventilated babies in the first 5 days of birth. Thirty-one preterm infants were enrolled, and tracheal aspirates taken until extubation to determine which levels of particular cytokines were abnormal in those with disease typical of “old” and “new” BPD. The eleven patients with both the old and new version of this disorder had elevated TGF-β1, though not statistically significant.(22)

### Tumor necrosis factor–α (TNF–α)

TNF-α is a proinflammatory cytokine that induces cell death, and enhances expression of other cytokines. Jonsson and colleagues enrolled 28 preterm infants, in a study to observe if early increased levels of proinflammatory cytokines predicted BPD. Their results showed TNF-α to be increased early in the seventeen patients who developed BPD.(27)

### Peptide growth factors

Peptide growth factors are important for normal lung development, maturation and repair. Inclusive of this group are fibroblast growth factor-2, vascular endothelial growth factor, endothelin-1 and keratinocyte growth factor.(52) Their specific roles are described below.

### Endothelin-1

Endothelin-1 is a potent endothelium-derived vasoconstricting and bronchoconstricting factor, produced in virtually all tissues, though its largest concentration is in the lung. Under the influence of cytokines (IL-1β, IL-6, IL-8), production of endothelins can be induced in macrophages and monocytes.(53) Endothelin-1 has also been hypothesized to increase the production of oxygen radicals from alveolar macrophages. It was theorized that in patients with BPD, that endothelin-1 would be up-regulated, secondary to increased inflammation. Thirty-four preterm infants with RDS were enrolled in a study to elucidate endothelin-1 levels and its association with BPD. Half the infants eventually developed BPD and in the first week of life it was noted that their endothelin-1 levels were significantly elevated when compared to infants who did not later develop BPD.(54)

### Fibroblast growth factor -2/basic fibroblast growth factor (FGF2/bFGF)

FGF plays a role in angiogenesis, by stimulating endothelial cell proliferation, degrading extracellular matrix and appears to interact with vascular endothelial growth factor.(55) Thirty premature infants were studied to evaluate levels of peptide growth factors, including FGF-2 and the outcome of BPD. In this study, 18 developed BPD and/or died. There was a significant elevation in the levels of FGF-2 in the BPD/death population on day 1 of life.(52)

### Keratinocyte growth factor (KGF)

A member of the fibroblast growth factor family, KGF regulates proliferation of alveolar epithelial cells, enhances synthesis of surfactant and accelerates wound closure in airway epithelium. Danan and colleagues evaluated levels of KGF in premature infants at risk for BPD. Ninety-one premature infants were sampled before day 5, while still intubated. Fourteen infants developed BPD. It was noted that in those patients who developed BPD, KGF was lower than in those who did not develop it.(56)

### Pulmonary hepatocyte growth factor (HGF)

HGF in fetal rat lungs stimulates branching of both alveolar and bronchial epithelia, and contributes to growth, maturation and maintenance of tissue homeostasis. It is suggested that HGF also mediates repair of type II pneumocytes after acute lung injury. Thirty two preterm infants were evaluated for levels of HGF and correlation with development of BPD. Seventeen out of 32 developed BPD and had significantly lower levels of HGF in the first 2 weeks of life, when compared to non-BPD counterparts.(57)

### Vascular endothelial growth factor (VEGF)

VEGF promotes endothelial cell growth and remodeling. In the pulmonary system it appears to be essential for the appropriate development of alveolar tissue; in rats its antagonism has been shown to result in dramatically impaired alveolarization, especially earlier in the course of lung development.(58) In heavily vascularized tissues, such as the lung, VEGF exists in high concentrations. During hyperoxic episodes when damage to the microvasculature occurs, VEGF plays a role in the remodeling process, and its’ levels are increased, sometimes disproportionately. As such, it was hypothesized that in BPD, VEGF levels may correlate with severity. Lassus and colleagues enrolled 44 preterm infants with RDS in a study to characterize VEGF levels in tracheal aspirates and plasma. They found that the 13 infants developing BPD had significantly lower levels of tracheal aspirate VEGF closer to one week of life.(59) No difference in tracheal aspirate VEGF levels were noted between BPD vs no BPD infants in the study by Ambalavanan et al.(52) In a recent study, a phasic pattern of VEGF concentrations was noted in infants who go on to develop BPD. These infants had an initial spike over the first 12 hours of postnatal life, followed by a decrease over the next few days and then a subsequent significant increase.(60)

## Summary

Bronchopulmonary dysplasia, a chronic respiratory disorder of very premature infants is one of the leading causes of mortality and morbidity in the neonatal intensive care unit. Currently there are many clinical associations predisposing to its’ development. Alterations in a variety of pathways involved in normal lung development have been proposed in the mechanisms responsible for the pathologic changes we observe with BPD. Inflammatory cells produce cytokines and chemoattractants for augmentation of the inflammatory response, in an attempt to curb the damage of the initial and potentially ongoing insults. Subsequent disruption of the alveolar capillary unit with increased vascular permeability, and decreased pulmonary tissue integrity occurs. The attempt at repair, differentiation and growth of these tissues act in concert with the inflammatory process to produce the biochemical markers, signs and symptoms observed in patients with, and those at risk for, BPD. As such, a variety of pulmonary biomarkers that relate intimately to these pathways have been studied to see if they predict which neonates will go on to develop the disorder. The aberrancies noted in the pulmonary concentrations of these biomarkers in preterm infants at risk for, or who have BPD, may one day be important for early diagnosis of the disorder. The limitations of the cited studies are the small sample size, and an inability to get biomarker concentrations in a control population of healthy, term infants. Currently, there is no one specific marker to predict development of BPD; thus we are limited to waiting for the clinical picture to take shape, often then managing its’ resultant morbidity and mortality. Perhaps there will ultimately be a panel of pulmonary biomarkers that indicate a strong predisposition prior to the clinical onset of BPD, or perhaps prior to its current time of diagnosis of 28 days postnatally or 36 weeks postmenstrually. This would allow us to be more targeted in our attempts at finding other therapies and modes of preventing this morbid sequela of prematurity.

## Figures and Tables

**Table 1:** Pulmonary biomarkers, proposed physiologic roles and alteration in BPD.

Biomarker	Physiologic role	Level in BPD
**Interleukins: Proinflammatory**		
IL-1β, IL-6, IL-16	Acute phase response	↑
IL-8/CXCL-8	Potent chemotactic agent for neutrophil recruitment	↑
**Interleukins: anti-inflammatory**		
IL10	Above + inhibits transcription factor NF-κβ whichlimits the inflammatory response	↓/↔
IL-4, IL-13	Production, differentiation and proliferation of B cells and macrophages. Also inhibits proinflammatory cytokine production	↔
Angiopoietin-2	Angiogenesis	↑
Cathepsin K	Protease	↓
CC Chemokines: MCP-1, 1α, 1β, 2 and 3	Inflammatory mediator	↑
Clara Cell Secretory Protein	Immunomodulator	↓
3-Chlorotyrosine	Marker of neutophil oxidation	↑
Fibronectin	Extracellular matrix formation	↑
Lysozyme	Bacteriocidal	↓
Lactoferrin	Antioxidant/anti-inflammatory	↓
Macrophage migration inhibitory factor	Inflammatory mediator	↓
Malondialdehyde	Oxidative byproduct	↑
Matrix Metalloproteinase-8	Protease (remodeling)	↑
NF-κβ	Transcription factor activated by signs of cellular stress	↑
Parathyroid Hormone-Related Protein	Alveolar growth/development	↓
Plasminogen Activator Inhibitor	Fibrinolysis	↑
Pulmonary Trypsin-2	Protease	↑
Soluble ICAM	Intercellular adhesion molecule for inflammatory, endothelial and bronchial epithelial cells	↑
Transforming Growth Factor- β1	Profibrotic	↑
Tumor Necrosis Factor –ά	Induces cell death, and enhances expression of other cytokines	↑
**Peptide Growth Factors**		
Endothelin-1	Vaso/bronchoconstrictor, proinflammatory	↑
Fibroblast Growth Factor-2	Angiogenic, endoproliferative	↑
Keratinocyte Growth Factor	Alveolar epithelial proliferation, repair	↓
Pulmonary Hepatocyte Growth Factor	Alveolar septation, maintenance, repair	↓
Vascular Endothelial Growth Factor	Angiogenesis	↑/↓
